# Supplementary material for: Functional analysis of a Phytophthora host-translocated effector using the yeast model system
Source: PeerJ. 2021 Dec 7;9:e12576. doi: 10.7717/peerj.12576 (PMC8663620; doi:10.7717/peerj.12576)
Supplement: Supplemental Information 2 — This R Notebook presents R code for analyzing the yeast growth inhibition data. [file peerj-09-12576-s002.pdf]

Data Analysis of Yeast Growth Inhibition Assays

William Morgan (College of Wooster)

Sep 11, 2020

This R Notebook presents R code for analyzing the yeast growth inhibition studies conducted by Matt Reeder (Wooster, ’13)

Prerequisites

The usual preliminary steps to prepare for analysis include clearing the Global Environment and loading needed R packages.

```
rm(list = ls())
library(tidyverse)

## ── Attaching packages ─────────────────────────────────────── tidyverse
1.3.0 ──

## ✓ ggplot2 3.3.0     ✓ purrr   0.3.4
## ✓ tibble  3.0.1     ✓ dplyr   0.8.5
## ✓ tidyr   1.0.3     ✓ stringr 1.4.0
## ✓ readr   1.3.1     ✓ forcats 0.5.0

## ── Conflicts ──────────────────────────────────────────
tidyverse_conflicts() ──
## x dplyr::filter() masks stats::filter()
## x dplyr::lag()    masks stats::lag()

library(multcomp) # note that this is masks dplyr::select

## Loading required package: mvtnorm

## Loading required package: survival

## Loading required package: TH.data

## Loading required package: MASS

##
## Attaching package: 'MASS'

## The following object is masked from 'package:dplyr':
##
##     select

##
## Attaching package: 'TH.data'
```

```
## The following object is masked from 'package:MASS':
##
##     geyser

library(modelr)
```

Yeast growth inhibition assays

The growth rate of *S. cerevisiae* yeast cells expressing a *P. sojae* RXLR effector was compared to those of control yeast cells (expressing a dummy gene, delta_GFP) grown at the same time (Experiments 4 and 5). The cell density of yeast cultures was estimated by measuring the optical density at 595 nm wavelength (OD595) at each timepoint (in hours).

Read and reformat the data

The time curve growth data for Matt Reeder's Set 4 and Set 5 results were copied into YeastGrowthData.csv, which we read in here. (If needed, the working directory should first be set to the folder containing both the .csv data file and this .Rmd Markdown notebook file.)

```
(growthData <- read_csv("YeastGrowthData.csv")
 )

## # A tibble: 30 x 11
##    Experiment Gene      Hour  Rep1  Rep2  Rep3  Rep4  Rep5  Rep6   Rep7
Rep8
##         <dbl> <chr>    <dbl> <dbl> <dbl> <dbl> <dbl> <dbl> <dbl>  <dbl>
<dbl>
##  1          4 dGFP      16.5 0.368 0.437 0.454 0.502 0.415 0.461  0.575
NA
##  2          4 dGFP      22.5 0.531 0.627 0.678 0.696 0.585 0.804  0.805
NA
##  3          4 dGFP      25   0.62  0.73  0.786 0.784 0.68  0.916  0.896
NA
##  4          4 PsAvr4_6  22.5 0.121 0.149 0.13  0.195 0.241 0.194 NA
NA
##  5          4 PsAvr4_6  25   0.136 0.179 0.147 0.23  0.28  0.216 NA
NA
##  6          4 PsAvr4_6  40.3 0.248 0.369 0.282 0.518 0.51  0.525 NA
NA
##  7          4 PsAvh172  22.5 0.129 0.138 0.109 0.124 0.139 0.121 NA
NA
##  8          4 PsAvh172  25   0.149 0.153 0.125 0.136 0.147 0.142 NA
NA
##  9          4 PsAvh172  40.3 0.259 0.235 0.208 0.2   0.24  0.262 NA
NA
## 10          4 PsAvh6    22.5 0.153 0.142 0.133 0.168 0.135 0.14  NA
```

```
NA
## # … with 20 more rows
```

The input data set contains 11 columns indicating the experiment, the expressed gene, the measurement hour, and the replicate number. We reformat the table to a longer format to facilitate subsequent data analysis and visualization and then add a column with the log transformation of OD595.

```
growthData <- growthData %>%
  pivot_longer(
    cols = starts_with("Rep"),
    names_to = "Replicate",
    values_to = "OD595",
    values_drop_na = TRUE
    )
growthData <- growthData %>%
  mutate(lnOD = log(OD595))
```

Let's collect the unique values for the Experiment, Gene and Replicate variables for later use.

```
valuesExperiment <- unique(growthData$Experiment)
valuesGene <- unique(growthData$Gene)
valuesReplicate <- unique(growthData$Replicate)
```

Estimate the slope of the best fit line and its fit

For each yeast culture (indicated by Experiment, Gene, Replicate), we determine its growth rate by computing a linear model of log transformed OD595 and hours of incubation. The estimated slope, b, of the best fit line is a measure of the growth rate, and r-squared is a measure of how well this line fits the data. Sample data with poorly fitting models, defined as r-squared less than 0.95, were omitted from further analysis.

```
cultureData <- tibble()
Experiment <- vector("double")
Gene <- vector("character")
Replicate <- vector("character")
Slope_Est <- vector("double")
Slope_SE <- vector("double")
R_squared <- vector("double")
Row <- 1
for(i in valuesExperiment) {
  for(j in valuesGene) {
    for(k in valuesReplicate) {
      cultureData <- growthData %>% filter(
        Experiment == i,
        Gene == j,
        Replicate == k
      )
      if(length(cultureData$lnOD > 0)) {
```

```
      regress_model <- lm(lnOD ~ Hour, data = cultureData)
      Experiment[Row] <- i
      Gene[Row] <- j
      Replicate[Row] <- k
      Slope_Est[Row] <- unname(regress_model$coefficients[2])
      Slope_SE[Row] <- summary(regress_model)$coefficients[2,2]
      R_squared[Row] <- summary(regress_model)$r.squared
      Row <- Row + 1
      }
    }
  }
}
slope_Rsq.Table <- tibble(
  Experiment,
  Gene,
  Replicate,
  Slope_Est,
  Slope_SE,
  R_squared
  )
(slope_Rsq.Table <- slope_Rsq.Table %>%
  filter(R_squared >= 0.95) %>%
   mutate(Gene = factor(Gene))
)

## # A tibble: 60 x 6
##    Experiment Gene     Replicate Slope_Est Slope_SE R_squared
##         <dbl> <fct>    <chr>         <dbl>    <dbl>     <dbl>
##  1          4 dGFP     Rep1         0.0613 0.000197     1.00
##  2          4 dGFP     Rep2         0.0603 0.000152     1.00
##  3          4 dGFP     Rep3         0.0650 0.00175      0.999
##  4          4 dGFP     Rep4         0.0528 0.00155      0.999
##  5          4 dGFP     Rep5         0.0579 0.000674     1.00
##  6          4 dGFP     Rep6         0.0830 0.00920      0.988
##  7          4 dGFP     Rep7         0.0529 0.00300      0.997
##  8          4 PsAvr4_6 Rep1         0.0399 0.000889     1.00
##  9          4 PsAvr4_6 Rep2         0.0497 0.00310      0.996
## 10          4 PsAvr4_6 Rep3         0.0432 0.000782     1.00
## # … with 50 more rows
```

Statistical tests for differential growth rates

Which of the RXLR effectors inhibit growth when expressed in yeast? The growth rates of each experimental strain expressing an RXLR effector will be compared to the control (dGFP) cultures grown at the same time (Experiment 4 vs. 5). Here we visualize the growth rates (that is, *b*) of each culture and show their average and 95% confidence interval for each strain.

```
slope_4.Table <- slope_Rsq.Table %>%
  filter(Experiment == 4)
```

```r
slope_4.Table %>%
  ggplot(aes(x=Gene, y=Slope_Est)) +
  geom_point(position=position_jitter(0.05), color = "red") +
  stat_summary(fun.data = "mean_cl_normal", mapping = aes(group = Gene)) +
  labs(x = "Expressed gene",
       y = "Growth rate (b)",
       title = "EXPERIMENT 4",
       caption = "Error bars are group means ± 95% confidence interval") +
  theme_classic(base_size = 13) +
  theme(legend.position = "none") +
  theme(axis.text.x=element_text(angle=30,hjust=1))
```

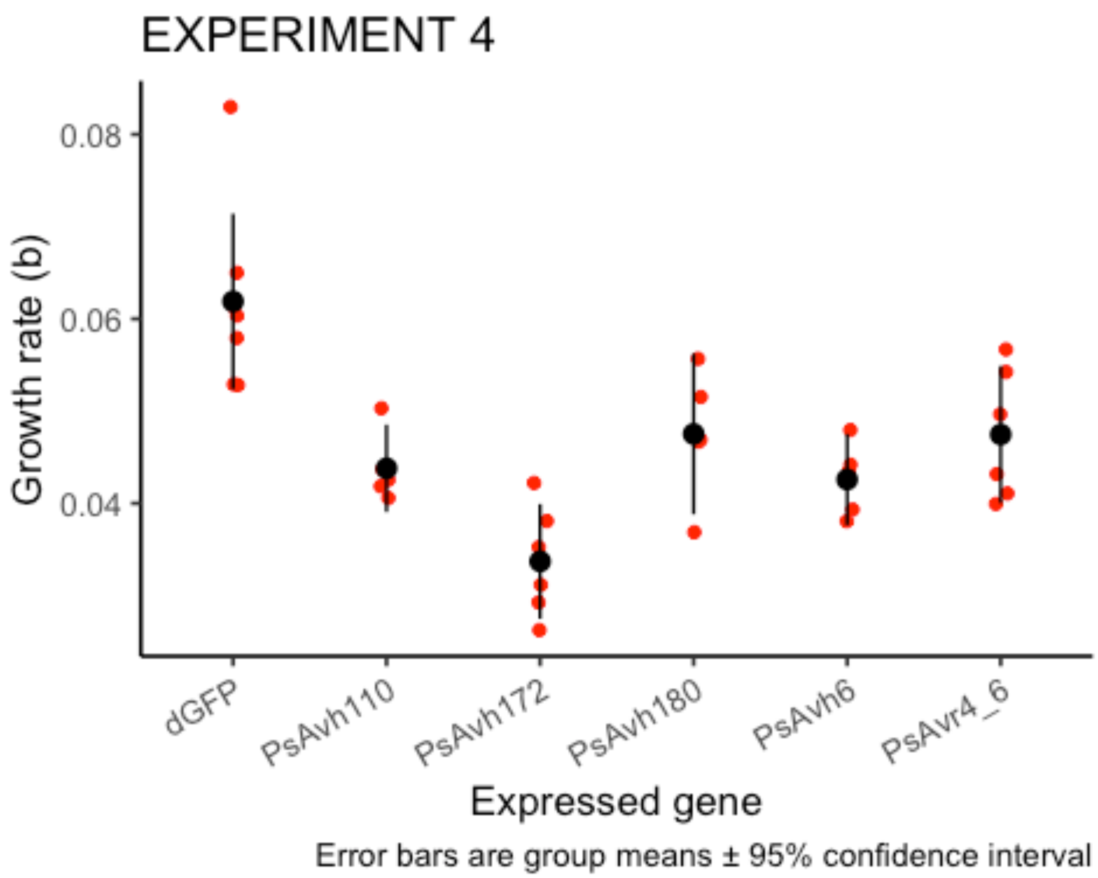


```r
slope_5.Table <- slope_Rsq.Table %>%
  filter(Experiment == 5)
slope_5.Table %>%
  ggplot(aes(x=Gene, y=Slope_Est)) +
  geom_point(position=position_jitter(0.05), color = "red") +
  stat_summary(fun.data = "mean_cl_normal", mapping = aes(group = Gene)) +
  labs(x = "Expressed gene",
       y = "Growth rate (b)",
       title = "EXPERIMENT 5",
       caption = "Error bars are group means ± 95% confidence interval") +
  theme_classic(base_size = 13) +
```

```
  theme(legend.position = "none") +
  theme(axis.text.x=element_text(angle=30,hjust=1))
```

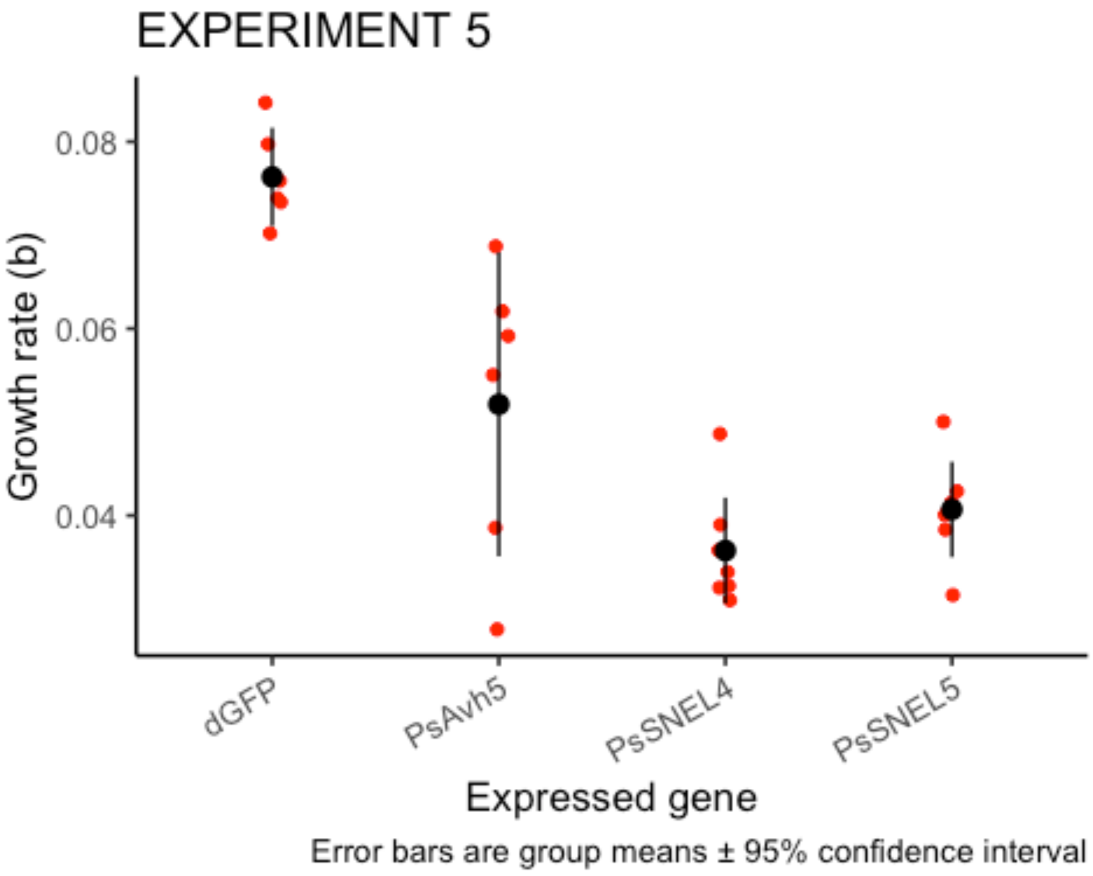


```
slope_Rsq.Table %>%
  ggplot(aes(x=Gene, y=Slope_Est)) +
  geom_point(position=position_jitter(0.05), color = "red") +
  stat_summary(fun.data = "mean_cl_normal", mapping = aes(group = Gene)) +
  labs(x = "Expressed gene",
       y = "Growth rate (b)",
       title = "BOTH EXPERIMENTS",
       caption = "Error bars are group means ± 95% confidence interval") +
  theme_classic(base_size = 13) +
  theme(legend.position = "none") +
  theme(axis.text.x=element_text(angle=30,hjust=1))
```

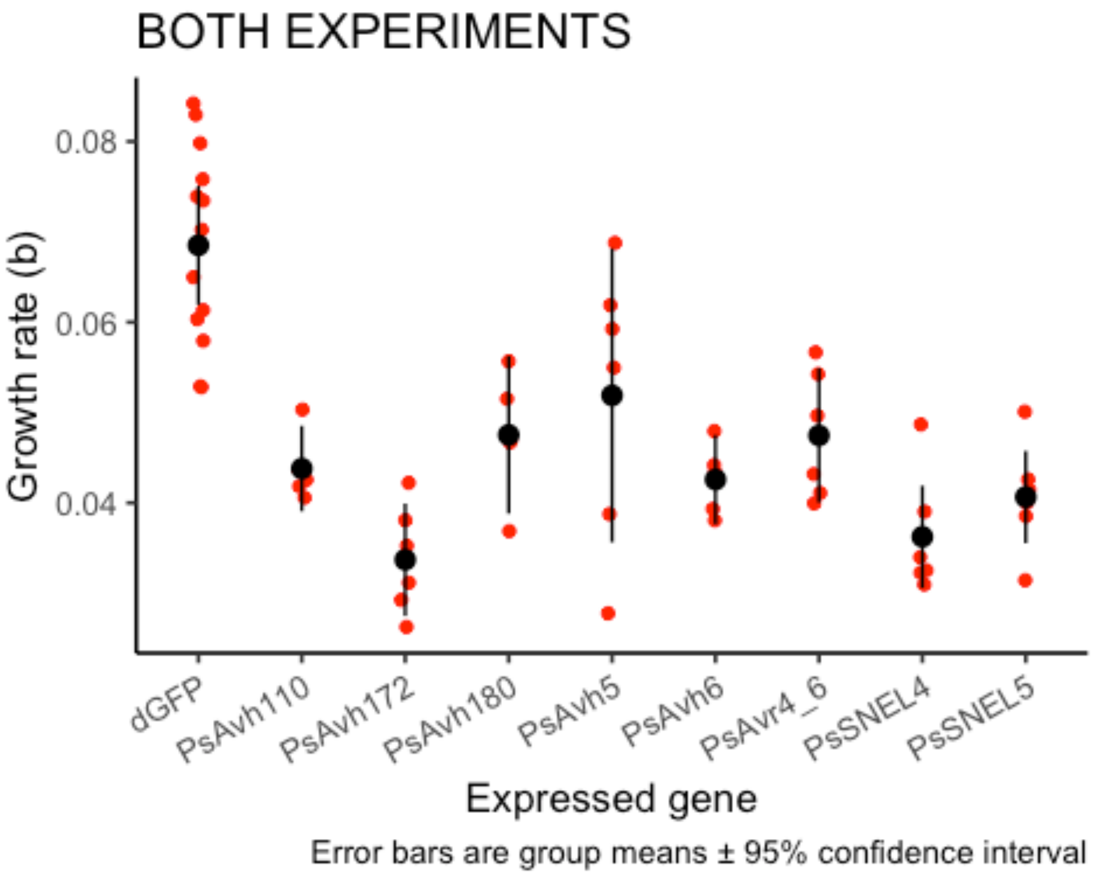

Next we conduct ANOVA to determine if any growth rates (b) significantly differ between the strains grown on the same day.

```
print("Experiment 4")

## [1] "Experiment 4"

anova(lm(Slope_Est ~ Gene, data = slope_4.Table))

## Analysis of Variance Table
##
## Response: Slope_Est
##           Df    Sum Sq    Mean Sq F value    Pr(>F)
## Gene       5 0.0027623 0.00055246  11.204 5.341e-06 ***
## Residuals 28 0.0013807 0.00004931
## ---
## Signif. codes:  0 '***' 0.001 '**' 0.01 '*' 0.05 '.' 0.1 ' ' 1

print("Experiment 5")

## [1] "Experiment 5"

anova(lm(Slope_Est ~ Gene, data = slope_5.Table))
```

```
## Analysis of Variance Table
##
## Response: Slope_Est
##           Df    Sum Sq    Mean Sq F value    Pr(>F)
## Gene       3 0.0060846 0.00202821  25.701 2.228e-07 ***
## Residuals 22 0.0017362 0.00007892
## ---
## Signif. codes:  0 '***' 0.001 '**' 0.01 '*' 0.05 '.' 0.1 ' ' 1
```

For both experiments, at least one strain had a growth rate that significantly differed from the others. Next we conduct planned comparisons of specific means using *t*-tests to determine which strains exhibit a significantly reduced growth rate relative to the control (dGFP).

```
print("Experiment 4")

## [1] "Experiment 4"

model <- lm(Slope_Est ~ Gene, data = slope_4.Table)
dGFP4_mean <- coef(model)[1] %>% unname() # for later use
PlannedTest <- glht(model=model, linfct = mcp(Gene = c("PsAvh110 - dGFP = 0",
                                                       "PsAvh172 - dGFP = 0",
                                                       "PsAvh180 - dGFP = 0",
                                                       "PsAvh6 - dGFP = 0",
                                                       "PsAvr4_6 - dGFP =
0")))
summary(PlannedTest)

##
##   Simultaneous Tests for General Linear Hypotheses
##
## Multiple Comparisons of Means: User-defined Contrasts
##
##
## Fit: lm(formula = Slope_Est ~ Gene, data = slope_4.Table)
##
## Linear Hypotheses:
##                       Estimate Std. Error t value Pr(>|t|)
## PsAvh110 - dGFP == 0 -0.018096   0.004112  -4.401  < 0.001 ***
## PsAvh172 - dGFP == 0 -0.028186   0.003907  -7.215  < 0.001 ***
## PsAvh180 - dGFP == 0 -0.014367   0.004112  -3.494  0.00728 **
## PsAvh6 - dGFP == 0   -0.019307   0.004112  -4.696  < 0.001 ***
## PsAvr4_6 - dGFP == 0 -0.014429   0.003907  -3.693  0.00425 **
## ---
## Signif. codes:  0 '***' 0.001 '**' 0.01 '*' 0.05 '.' 0.1 ' ' 1
## (Adjusted p values reported -- single-step method)

print("Experiment 5")

## [1] "Experiment 5"
```

```
model <- lm(Slope_Est ~ Gene, data = slope_5.Table)
dGFP5_mean <- coef(model)[1] %>% unname() # for later use
PlannedTest <- glht(model=model, linfct = mcp(Gene = c("PsAvh5 - dGFP = 0",
                                                       "PsSNEL4 - dGFP = 0",
                                                       "PsSNEL5 - dGFP =
0")))
summary(PlannedTest)
```

```
##
##   Simultaneous Tests for General Linear Hypotheses
##
## Multiple Comparisons of Means: User-defined Contrasts
##
##
## Fit: lm(formula = Slope_Est ~ Gene, data = slope_5.Table)
##
## Linear Hypotheses:
##                       Estimate Std. Error t value Pr(>|t|)
## PsAvh5 - dGFP == 0  -0.024335   0.005129  -4.745 0.000228 ***
## PsSNEL4 - dGFP == 0 -0.039985   0.004942  -8.090  < 1e-04 ***
## PsSNEL5 - dGFP == 0 -0.035583   0.004942  -7.200  < 1e-04 ***
## ---
## Signif. codes:  0 '***' 0.001 '**' 0.01 '*' 0.05 '.' 0.1 ' ' 1
## (Adjusted p values reported -- single-step method)
```

```
print("Combined Experiments")
```

```
## [1] "Combined Experiments"
```

```
slope.Table <- slope_Rsq.Table
model <- lm(Slope_Est ~ Gene, data = slope.Table)
dGFPall_mean <- coef(model)[1] %>% unname() # for later use
PlannedTest <- glht(model=model, linfct = mcp(Gene = c("PsAvh110 - dGFP = 0",
                                                       "PsAvh172 - dGFP = 0",
                                                       "PsAvh180 - dGFP = 0",
                                                       "PsAvh6 - dGFP = 0",
                                                       "PsAvr4_6 - dGFP = 0",
                                                       "PsAvh5 - dGFP = 0",
                                                       "PsSNEL4 - dGFP = 0",
                                                       "PsSNEL5 - dGFP =
0")))
summary(PlannedTest)
```

```
##
##   Simultaneous Tests for General Linear Hypotheses
##
## Multiple Comparisons of Means: User-defined Contrasts
##
##
## Fit: lm(formula = Slope_Est ~ Gene, data = slope.Table)
##
```

```
## Linear Hypotheses:
##                          Estimate Std. Error t value Pr(>|t|)
## PsAvh110 - dGFP == 0 -0.024714   0.004531  -5.454  < 1e-04 ***
## PsAvh172 - dGFP == 0 -0.034805   0.004250  -8.190  < 1e-04 ***
## PsAvh180 - dGFP == 0 -0.020986   0.004531  -4.631 0.000198 ***
## PsAvh6 - dGFP == 0   -0.025926   0.004531  -5.722  < 1e-04 ***
## PsAvr4_6 - dGFP == 0 -0.021047   0.004250  -4.953  < 1e-04 ***
## PsAvh5 - dGFP == 0   -0.016613   0.004250  -3.909 0.002125 **
## PsSNEL4 - dGFP == 0  -0.032264   0.004037  -7.993  < 1e-04 ***
## PsSNEL5 - dGFP == 0  -0.027862   0.004037  -6.902  < 1e-04 ***
## ---
## Signif. codes:  0 '***' 0.001 '**' 0.01 '*' 0.05 '.' 0.1 ' ' 1
## (Adjusted p values reported -- single-step method)
```

Visual comparison of normalized growth rates

The growth rate was normalized by dividing b of each experimental culture by the mean value of b of control (ΔGFP) cultures grown at the same time. The normalized growth rate of all strains can then be compared on a single plot.

```
slope_4.Table <- slope_4.Table %>%
  mutate(Normalized_b = Slope_Est / dGFP4_mean)
slope_5.Table <- slope_5.Table %>%
  mutate(Normalized_b = Slope_Est / dGFP5_mean)
slope_Rsq.Table <- rbind(slope_4.Table, slope_5.Table)

slope_Rsq.Table %>%
  ggplot(aes(x=Gene, y=Normalized_b)) +
  geom_point(position=position_nudge(0.2), color = "red") +
  stat_summary(fun.data = "mean_cl_normal", mapping = aes(group = Gene)) +
  labs(x = "Expressed gene", y = "Normalized growth rate") +
  theme_classic(base_size = 13) +
  theme(legend.position = "none") +
  annotate(geom = "text", x = slope_Rsq.Table$Gene, y = 0.99, label = "*",
size = 7) +
  theme(axis.text.x=element_text(angle=30,hjust=1))
```

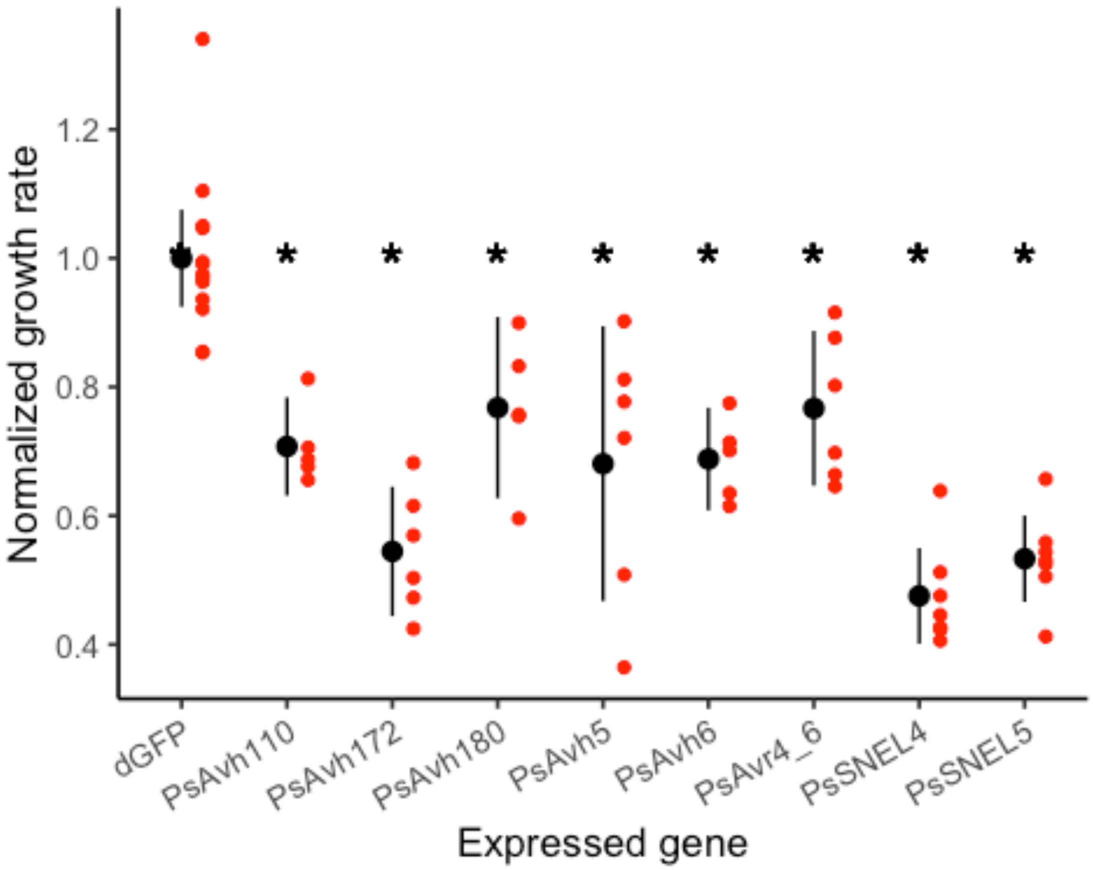

Figure. Normalized growth rates of yeast strains expressing individual *P. sojae* RXLR effectors. The normalized growth rate of individual cultures is marked in red; the mean and 95% confidence interval of each strain is indicated by a black dot and vertical lines. Asterisks indicate cultures with significantly reduced mean growth rates (adjusted p-values < 0.01).
