# Supplementary material for: Functional analysis of a Phytophthora host-translocated effector using the yeast model system
Source: PeerJ. 2021 Dec 7;9:e12576. doi: 10.7717/peerj.12576 (PMC8663620; doi:10.7717/peerj.12576)
Supplement: Supplemental Information 3 — In this R notebook, we present code to identify publicly available yeast transcriptome profiles similar to that of PsAvh110-expressing yeast cells. [file peerj-09-12576-s003.pdf]

Search for matching transcriptome profiles

William Morgan (College of Wooster)

October 2020

One approach to examine the possible targets of a pathogen effector in yeast is to identify perturbations that produce a comparable transcriptome response. Our RNA-seq analysis indicates that *S. cerevisiae* overexpressing *P. sojae* Avh110 exhibit a >2-fold reduction in mRNA levels of five genes related to phosphate metabolism. In this R notebook, we searched transcriptome profiles available in publicly availalable databases to identify other perturbations where expression of these five genes are among the most significantly affected.

Prerequisites

The usual preliminary steps to prepare for analysis include clearing the Global Environment and loading needed R packages.

```
rm(list = ls())
library(biomaRt)
library(tidyverse)
```

Retrieve list of yeast genes that are most repressed by PsAvh110 overexpression

After loading our PsAvh110 RNA-seq data set, the genes are sorted by log2-fold change and then the list of yeast genes with a >2-fold reduction (lfc < -1.0) in mRNA levels are retrieved. The gene names for each gene ID are also retrieved from Ensembl's BioMart.

```
# EMTAB9566_processed <- read_csv("https://www.ebi.ac.uk/arrayexpress/files/E
-MTAB-9566/DESeq2results_PsAvh110vs.dGFP.csv", col_names = FALSE)
EMTAB9566_processed <- read_csv("Galaxy32-(DESeq2_results_PsAvh110).csv")
EMTAB9566_processed <- EMTAB9566_processed %>% arrange(`log2(FC)`)
BottomGenes <- EMTAB9566_processed %>%
  filter(`log2(FC)` < -1.0) %>% pull(GeneID)

BottomGeneTable<- getBM(attributes = c("external_gene_name", "ensembl_gene_id
"),
      filters = c("ensembl_gene_id"),
      values = BottomGenes,
      mart = useMart(biomart = "ensembl",
                     dataset = "scerevisiae_gene_ensembl",
                     host = "useast.ensembl.org"))
BottomGeneTable <- BottomGeneTable %>%
  select(ORF = ensembl_gene_id, Gene_name = external_gene_name)
BottomGeneTable
```

```
##       ORF Gene_name
## 1 YBR093C      PHO5
## 2 YBR296C     PHO89
## 3 YHR136C      SPL2
## 4 YHR215W     PHO12
## 5 YML123C     PHO84
```

GSE5499 data set

Chua et al. (2006; PNAS PMID: 16880382) analyzed 55 TF overexpression strains and 51 non-essential TF deletion mutants by microarray with fluor-reversal replicates. Details about each GSM sample are retrieved below.

```
# Table with details about each GSM sample
GSE5499_GSMinfo <- read_tsv("https://ftp.ncbi.nlm.nih.gov/geo/series/GSE5nnn/
GSE5499/matrix/GSE5499_series_matrix.txt.gz", col_names = FALSE, skip = 36, n
_max = 19)
# get labels in first column (without !) and use as column names in transpose
d table
labels <- gsub("!", "", GSE5499_GSMinfo$X1)
GSE5499_GSMinfo <- GSE5499_GSMinfo %>% t()
colnames(GSE5499_GSMinfo) <- labels
# convert to tibble, slice out first row, select desired columns & extract ge
notype
GSE5499_GSMinfo <- GSE5499_GSMinfo %>%
  as_tibble() %>%
  slice(-1) %>%
  select(Sample_geo_accession, Sample_description,
         Sample_source_name_ch1, Sample_source_name_ch2) %>%
  mutate(Mutant_genotype = str_extract(Sample_description, boundary("word")))
```

We will analyze this data set to see which transcriptome profiles exhibits a similar repression of the PsAvh110 most-repressed genes. Before downloading the normalized log ratios for each microarray feature, we retrieve a table allowing us to substitute ORF names (e.g., YOR348C) for the microarray feature IDs (e.g., #1).

```
# Table with each feature ID and its ORF
GSE5499_ORFs <- read_table2("https://ftp.ncbi.nlm.nih.gov/geo/series/GSE5nnn/
GSE5499/soft/GSE5499_family.soft.gz", skip = 344, n_max = 6307)

# Download normalized log ratios, replace ID_REFs with ORFs & make variables
numeric
GSE5499_matrix <- read_tsv("https://ftp.ncbi.nlm.nih.gov/geo/series/GSE5nnn/G
SE5499/matrix/GSE5499_series_matrix.txt.gz", comment = "!")
GSE5499_matrix <- GSE5499_ORFs %>%
  select("ORF") %>%
  cbind(GSE5499_matrix) %>%
  select(-ID_REF)
GSE5499_matrix <- GSE5499_matrix %>%
  mutate_at(-1, funs(as.numeric))
```

Correcting replicate 2 ratios

The data matrix contains normalized log ch2/ch1 ratios for each sample. Because these are dye-swap microarray experiments, replicate 1 has the mutant/control ratio, while replicate 2 has the control/mutant ratio. To correct this, the replicate 2 ratios were inverted by multiplying the log values by -1.

```
GSMs_rep2 <- GSE5499_GSMinfo %>%
  filter(str_detect(Sample_description, "replicate 2")) %>%
  pull(Sample_geo_accession)
inverse_sign <- function(x, na.rm = FALSE) (x * -1)
GSE5499_corrected <- GSE5499_matrix %>%
  mutate_at(GSMs_rep2, inverse_sign)

# transform this data to long format for plotting later
GSE5499_longer <- GSE5499_corrected %>%
  pivot_longer(-ORF, names_to = "Sample_geo_accession", values_to = "log_rati
o")
```

Screen for samples with a similar repression pattern

To compare each transcriptome profile to the PsAvh110-repressed gene set, we rank each gene's expression level within each GSM sample, where #1 has the most negative value. After extracting all rankings for the PsAvh110-repressed genes, we then identify those GSM samples with the lowest mean of log-transformed rankings for these genes (that is, greatest reduction in expression on average). The log-transformation reduces the impact of higher ranked genes, which differ negligibly in expression levels. To reduce the effects of missing data (which can skew the mean), we only retain samples with expression data for at least three of the five PsAvh110-repressed genes.

```
GSE5499_rank <- GSE5499_corrected  %>%
  mutate_if(is.numeric, funs(min_rank))

GSE5499_rank_BottomGenes <- GSE5499_rank %>% filter(ORF %in% BottomGenes)

GSE5499_mean_logrank_BottomGenes <- GSE5499_rank_BottomGenes %>%
  pivot_longer(cols = -1, names_to = "Sample_geo_accession", values_to = "Sam
ple_rank") %>%
    filter(!is.na(Sample_rank)) %>%
  group_by(Sample_geo_accession) %>%
  summarise(mean_logrank = 10^mean(log10(Sample_rank)), n = n()) %>%
  filter(n > 2) %>%
  arrange(mean_logrank)

## `summarise()` ungrouping output (override with `.groups` argument)

GSE5499_mean_logrank_BottomGenes
```

```
## # A tibble: 264 x 3
##    Sample_geo_accession mean_logrank     n
##    <chr>                       <dbl> <int>
##  1 GSM126823                    17.6     5
##  2 GSM126822                    18.4     5
##  3 GSM126837                    20.0     4
##  4 GSM126866                    27.4     5
##  5 GSM126867                    36.9     5
##  6 GSM126836                    39.1     5
##  7 GSM126793                    42.6     5
##  8 GSM126743                    64.9     4
##  9 GSM126792                    70.9     5
## 10 GSM126739                    94.7     4
## # … with 254 more rows
```

To examine the most interesting sample data more closely, we display the rankings of the PsAvh110-repressed genes for the GSM samples with the lowest mean_logranks (Bonferroni-adjusted p-value < 0.001; see Appendix below), only retaining those GSM samples where both members of the dye-swap pair have low mean_logranks (to ensure reproducibility).

```
GSE5499_lo_mr_BG <- GSE5499_mean_logrank_BottomGenes %>%
  filter(mean_logrank < 100) %>%
  left_join(GSE5499_GSMinfo, by = "Sample_geo_accession") %>%
  arrange(Sample_geo_accession) %>%
  mutate(Paired = (Mutant_genotype == lag(Mutant_genotype)) |
           (Mutant_genotype == lead(Mutant_genotype)))

GSE5499_lowestGSMpairs <- GSE5499_lo_mr_BG %>%
  filter(Paired == TRUE) %>%
  arrange(mean_logrank) %>%
  pull(Sample_geo_accession)

lowestGSM_rank_BG <- GSE5499_rank_BottomGenes %>%
  select(ORF, all_of(GSE5499_lowestGSMpairs))

GSE5499_longer_BG <- GSE5499_longer %>%
  filter(ORF %in% BottomGenes)
GSE5499_longer_NBG <- GSE5499_longer %>%
  filter(!ORF %in% BottomGenes)

Plot_data <- lowestGSM_rank_BG %>%
  pivot_longer(-ORF, names_to = "Sample_geo_accession", values_to = "Rank_in_
sample") %>%
  mutate(Sample_geo_accession = toupper(Sample_geo_accession)) %>%
  left_join(GSE5499_longer_BG, by = c("ORF", "Sample_geo_accession"))

Plot_data %>%
  ggplot(aes(x=Sample_geo_accession, y=log_ratio)) +
  geom_point(data=filter(GSE5499_longer, !ORF %in% BottomGenes,
```

```
                              Sample_geo_accession %in% GSE5499_lowestGSMpairs,
                              log_ratio < 0) %>%
                  mutate(Sample_geo_accession = factor(Sample_geo_accession,
                                                        levels = GSE5499_lowestGS
Mpairs)),
               na.rm = TRUE,
               position=position_jitter(0.1,0), color="black", alpha = 0.2) +
  geom_label(aes(label=Rank_in_sample, color = ORF),
               position = position_dodge2(width = 0.6),
              label.padding = unit(0.05, "lines"),
              na.rm = TRUE,
              show.legend = FALSE) +
  geom_label(data = GSE5499_mean_logrank_BottomGenes %>%
                filter(Sample_geo_accession %in% GSE5499_lowestGSMpairs),
              aes(label=signif(mean_logrank,3), y=min(Plot_data$log_ratio, na.r
m = TRUE)),
              nudge_y = -0.1,
              label.padding = unit(0.10, "lines"),
              show.legend = FALSE) +
  geom_text(data = GSE5499_GSMinfo %>%
                filter(Sample_geo_accession %in% toupper(GSE5499_lowestGSMpair
s)),
              aes(label=Mutant_genotype, y=min(Plot_data$log_ratio, na.rm = TRU
E)),
              nudge_y = -0.2,
              show.legend = FALSE) +
  geom_point(aes(color = ORF), size=NA, na.rm = TRUE) + # to replace geom_lab
el legend symbols
  labs(x = NULL,
       y = "Normalized log2 ratio (mutant/control)") +
  theme_classic(base_size = 13) +
  theme(axis.text.x=element_text(angle=30,hjust=1)) +
  scale_y_continuous(limits = c(min(Plot_data$log_ratio)-2, 0)) +
  scale_color_discrete(name = "Gene name", labels = BottomGeneTable$Gene_name
) +
  guides(colour=guide_legend(override.aes=list(size=3)))
```

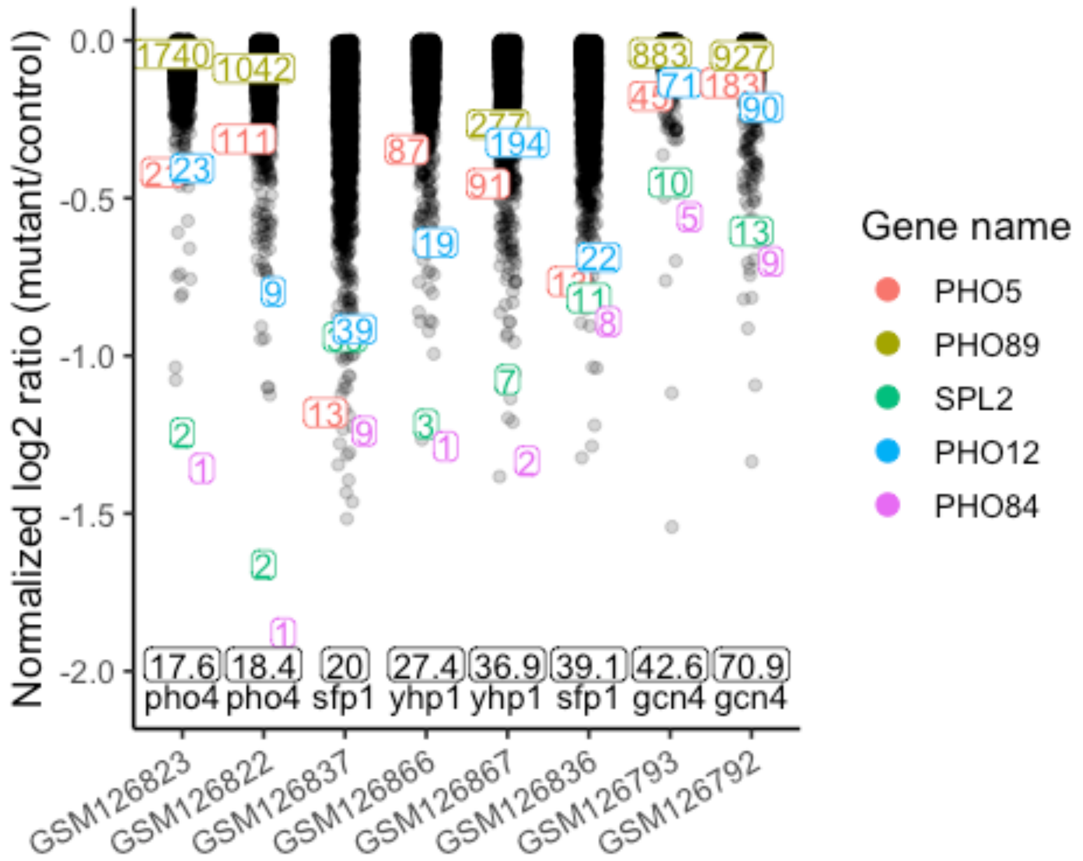

Figure S2-1. Expression levels and sample ranks of the PsAvh110-repressed genes in mutant samples with lowest mean rank. For each sample, the relative expression levels of the five PsAvh110-repressed genes are indicated by a colored box labeled with their rank position. The relative expression levels of all other yeast genes are indicated by gray dots. The mean of the gene ranks (after removing missing data) and the mutant genotype of each sample is indicated below. Overexpression mutants are indicated in all caps; deletion mutants in lower case.

```
MutantDescriptions<- getBM(attributes = c("external_gene_name", "description"
),
      filters = c("external_gene_name"),
      values = GSE5499_GSMinfo %>%
        filter(Sample_geo_accession %in% toupper(GSE5499_lowestGSMpairs)) %>%
        pull(Mutant_genotype) %>% unique() %>% toupper(),
      mart = useDataset("scerevisiae_gene_ensembl", mart = useMart("ensembl")
))
MutantDescriptions

##   external_gene_name
## 1               GCN4
## 2               PHO4
## 3               SFP1
## 4               YHP1
##
```

```
description
## 1
bZIP transcriptional activator of amino acid biosynthetic genes; activator re
sponds to amino acid starvation; expression is tightly regulated at both the
transcriptional and translational levels [Source:SGD;Acc:S000000735]
## 2
Basic helix-loop-helix (bHLH) transcription factor of the myc-family; activat
es transcription cooperatively with Pho2p in response to phosphate limitation
; binding to 'CACGTG' motif is regulated by chromatin restriction, competitiv
e binding of Cbf1p to the same DNA binding motif and cooperation with Pho2p;
function is regulated by phosphorylation at multiple sites and by phosphate a
vailability [Source:SGD;Acc:S000001930]
## 3 Regulates transcription of ribosomal protein and biogenesis genes; regul
ates response to nutrients and stress, G2/M transitions during mitotic cell c
ycle and DNA-damage response, and modulates cell size; regulated by TORC1 and
Mrs6p; sequence of zinc finger, ChIP localization data, and protein-binding m
icroarray (PBM) data, and computational analyses suggest it binds DNA directl
y at highly active RP genes and indirectly through Rap1p at others; can form
the [ISP+] prion [Source:SGD;Acc:S000004395]
## 4
Homeobox transcriptional repressor; binds Mcm1p and early cell cycle box (ECB
) elements of cell cycle regulated genes, thereby restricting ECB-mediated tr
anscription to the M/G1 interval; YHP1 has a paralog, YOX1, that arose from t
he whole genome duplication [Source:SGD;Acc:S000002859]
```

Screening for samples with the inverse pattern

It might also be informative to identify mutants that exhibit the opposite pattern; that is, the PsAvh110-repressed genes show the greatest increases in relative expression levels. We perform this inverted ranking by inverting the normalized log ratios prior to ranking. To distinguish the inverted rankings from the previous rankings, the samples are renamed in lowercase (“gsm”).

```
GSE5499_rank2 <- GSE5499_corrected  %>%
  rename_if(is.numeric, tolower) %>%
  mutate_if(is.numeric, inverse_sign) %>%
  mutate_if(is.numeric, funs(min_rank))

GSE5499_rank2_BottomGenes <- GSE5499_rank2 %>% filter(ORF %in% BottomGenes)

GSE5499_mean_logrank2_BottomGenes <- GSE5499_rank2_BottomGenes %>%
  pivot_longer(cols = -1, names_to = "Sample_geo_accession", values_to = "Sam
ple_rank") %>%
    filter(!is.na(Sample_rank)) %>%
  group_by(Sample_geo_accession) %>%
  summarise(mean_logrank = 10^mean(log10(Sample_rank)), n = n()) %>%
  filter(n > 2) %>%
  arrange(mean_logrank)

## `summarise()` ungrouping output (override with `.groups` argument)
```

```r
GSE5499_mean_logrank2_BottomGenes

## # A tibble: 264 x 3
##    Sample_geo_accession mean_logrank     n
##    <chr>                       <dbl> <int>
##  1 gsm126661                    2.21     4
##  2 gsm126660                    4.10     5
##  3 gsm126778                   12.0      3
##  4 gsm126833                   13.5      3
##  5 gsm126779                   14.2      3
##  6 gsm126774                   38.8      5
##  7 gsm126775                   46.2      4
##  8 gsm126802                   59.4      5
##  9 gsm126803                   80.2      5
## 10 gsm126610                   88.6      3
## # … with 254 more rows

GSE5499_GSMinfo <- GSE5499_GSMinfo %>%
  mutate(Sample_geo_accession = tolower(Sample_geo_accession)) %>%
  bind_rows(GSE5499_GSMinfo, .)

GSE5499_lo_mr2_BG <- GSE5499_mean_logrank2_BottomGenes %>%
  filter(mean_logrank < 100) %>%
  left_join(GSE5499_GSMinfo, by = "Sample_geo_accession") %>%
  arrange(Sample_geo_accession) %>%
  mutate(Paired = (Mutant_genotype == lag(Mutant_genotype)) |
           (Mutant_genotype == lead(Mutant_genotype)))

GSE5499_lowestGSM2pairs <- GSE5499_lo_mr2_BG %>%
  filter(Paired == TRUE) %>%
  arrange(mean_logrank) %>%
  pull(Sample_geo_accession)

lowestGSM2_rank_BG <- GSE5499_rank2_BottomGenes %>%
  select(ORF, all_of(GSE5499_lowestGSM2pairs))

lowestGSM2_rank_BG %>%
  pivot_longer(-1, names_to = "Sample_geo_accession", values_to = "Rank") %>%
  filter(!is.na(Rank)) %>%
  count(Sample_geo_accession) %>%
  filter(n > 3)

## # A tibble: 6 x 2
##   Sample_geo_accession     n
##   <chr>                <int>
## 1 gsm126660                5
## 2 gsm126661                4
## 3 gsm126774                5
## 4 gsm126775                4
```

```
## 5 gsm126802                5
## 6 gsm126803                5
```

```
Plot_data2 <- lowestGSM2_rank_BG %>%
  pivot_longer(-ORF, names_to = "Sample_geo_accession", values_to = "Rank_in_
sample") %>%
  mutate(Sample_geo_accession = toupper(Sample_geo_accession)) %>%
  left_join(GSE5499_longer_BG, by = c("ORF", "Sample_geo_accession"))

Plot_data2 %>%
  ggplot(aes(x=Sample_geo_accession, y=log_ratio)) +
  geom_point(data=filter(GSE5499_longer, !ORF %in% BottomGenes,
                         Sample_geo_accession %in% toupper(GSE5499_lowestGSM2
pairs),
                         log_ratio > 0) %>%
               mutate(Sample_geo_accession = factor(Sample_geo_accession,
                                                    levels = toupper(GSE5499_
lowestGSM2pairs))),
             na.rm = TRUE,
             position=position_jitter(0.1,0), color="black", alpha = 0.2) +
  geom_label(aes(label=Rank_in_sample, color = ORF),
             position = position_dodge2(width = 0.6),
            label.padding = unit(0.05, "lines"),
            na.rm = TRUE,
            show.legend = FALSE) +
  geom_label(data = GSE5499_mean_logrank2_BottomGenes %>%
               mutate(Sample_geo_accession = toupper(Sample_geo_accession)) %
>%
               filter(Sample_geo_accession %in% toupper(GSE5499_lowestGSM2pai
rs)),
            aes(label=signif(mean_logrank,3), y=0),
            nudge_y = -0.1,
            label.padding = unit(0.10, "lines"),
            show.legend = FALSE) +
  geom_text(data = GSE5499_GSMinfo %>%
               filter(Sample_geo_accession %in% toupper(GSE5499_lowestGSM2pai
rs)),
            aes(label=Mutant_genotype, y=0),
            nudge_y = -0.2,
            show.legend = FALSE) +
  geom_point(aes(color = ORF), size=NA, na.rm = TRUE) + # to replace geom_lab
el legend symbols
  labs(x = NULL,
       y = "Normalized log2 ratio (mutant/control)") +
  theme_classic(base_size = 13) +
  theme(axis.text.x=element_text(angle=30,hjust=1)) +
  scale_y_continuous(limits = c(-0.2, NA)) +
  scale_color_discrete(name = "Gene name", labels = BottomGeneTable$Gene_name
) +
  guides(colour=guide_legend(override.aes=list(size=3)))
```

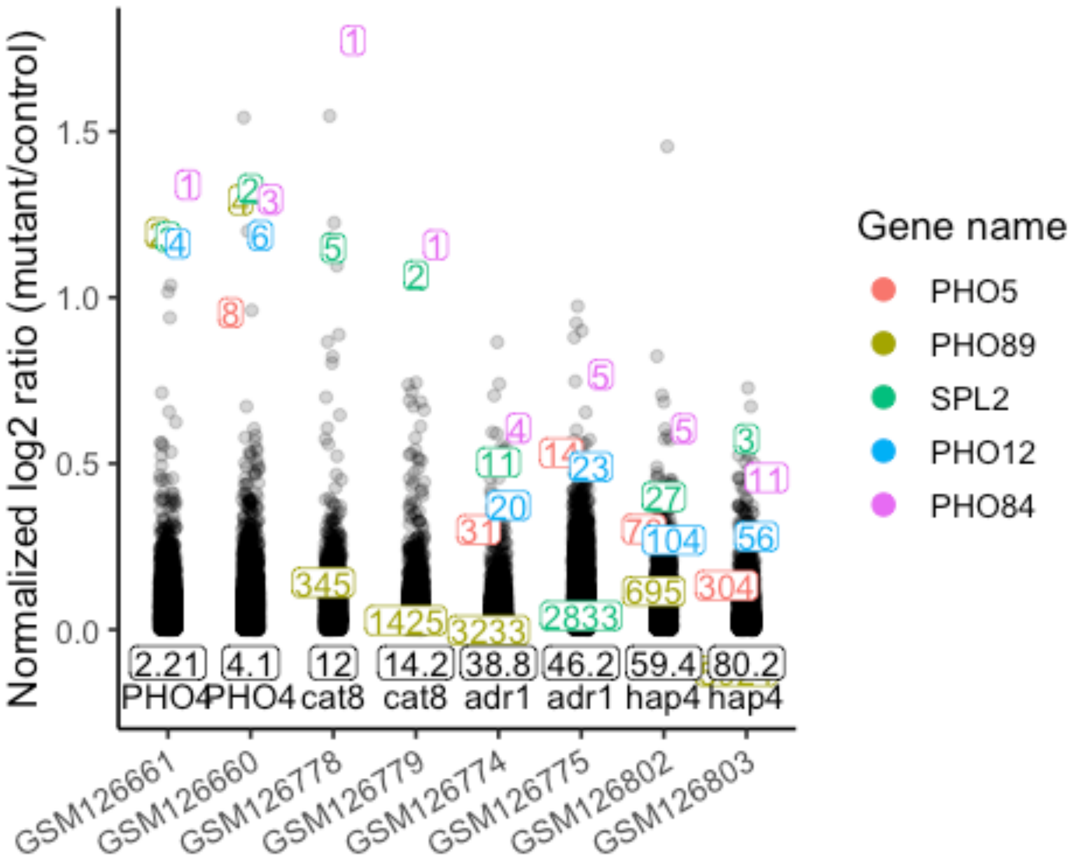

Figure S2-2. Expression levels and sample ranks of the PsAvh110-repressed genes in mutant samples with lowest inverse mean rank. For each sample, the relative expression levels of the five PsAvh110-repressed genes (ORFs) are indicated by a colored box labeled with their inverse rank position. The relative expression levels of all other yeast genes are indicated by gray dots. The mean of the inverse ranks (after removing missing data) and the mutant genotype of each sample is indicated below. Overexpression mutants are indicated in all caps; deletion mutants in lower case.

```
MutantDescriptions2<- getBM(attributes = c("external_gene_name", "description
"),
      filters = c("external_gene_name"),
      values = GSE5499_GSMinfo %>%
        filter(Sample_geo_accession %in% toupper(GSE5499_lowestGSM2pairs)) %>
%
        pull(Mutant_genotype) %>% unique() %>% toupper(),
      mart = useDataset("scerevisiae_gene_ensembl", mart = useMart("ensembl")
))
MutantDescriptions2
```

```
##   external_gene_name
## 1               ADR1
## 2               CAT8
## 3               HAP4
```

```
## 4                PHO4
##
description
## 1
Carbon source-responsive zinc-finger transcription factor; required for trans
cription of the glucose-repressed gene ADH2, of peroxisomal protein genes, an
d of genes required for ethanol, glycerol, and fatty acid utilization [Source
:SGD;Acc:S000002624]
## 2
Zinc cluster transcriptional activator; necessary for derepression of a varie
ty of genes under non-fermentative growth conditions, active after diauxic sh
ift, binds carbon source responsive elements; relative distribution to the nu
cleus increases upon DNA replication stress [Source:SGD;Acc:S000004893]
## 3
Transcription factor; subunit of the heme-activated, glucose-repressed Hap2p/
3p/4p/5p CCAAT-binding complex, a transcriptional activator and global regula
tor of respiratory gene expression; provides the principal activation functio
n of the complex; involved in diauxic shift [Source:SGD;Acc:S000001592]
## 4 Basic helix-loop-helix (bHLH) transcription factor of the myc-family; ac
tivates transcription cooperatively with Pho2p in response to phosphate limit
ation; binding to 'CACGTG' motif is regulated by chromatin restriction, compe
titive binding of Cbf1p to the same DNA binding motif and cooperation with Ph
o2p; function is regulated by phosphorylation at multiple sites and by phosph
ate availability [Source:SGD;Acc:S000001930]
```

GSE97799 data set

He BZ, Zhou X, O'Shea EK. Evolution of reduced co-activator dependence led to target expansion of a starvation response pathway. Elife 2017 May 9;6. PMID: 28485712. The goal is to identify genes induced by each of the eight Pho4 orthologs in the S. cerevisiae background, either with or without S. cerevisiae Pho2 (ScPho2) and with the negative regulator of Pho4, Pho80, deleted.

Details about each GSM sample are retrieved below.

```
# Table with details about each GSM sample
GSE97799_series_matrix_txt <- read_delim("https://ftp.ncbi.nlm.nih.gov/geo/se
ries/GSE97nnn/GSE97799/matrix/GSE97799_series_matrix.txt.gz", "\t", escape_do
uble = FALSE, trim_ws = TRUE, skip = 32)
GSE97799_series_matrix_txt
```

```
## # A tibble: 45 x 37
##    `!Sample_geo_ac… GSM2577557 GSM2577558 GSM2577559 GSM2577560 GSM2577561
##    <chr>            <chr>      <chr>      <chr>      <chr>      <chr>
##  1 !Sample_status   Public on… Public on… Public on… Public on… Public on…
##  2 !Sample_submiss… Apr 14 20… Apr 14 20… Apr 14 20… Apr 14 20… Apr 14 20…
##  3 !Sample_last_up… May 15 20… May 15 20… May 15 20… May 15 20… May 15 20…
##  4 !Sample_type     SRA        SRA        SRA        SRA        SRA
##  5 !Sample_channel… 1          1          1          1          1
##  6 !Sample_source_… OD600=0.3… OD600=0.3… OD600=0.3… OD600=0.3… OD600=0.3…
```

```
##  7 !Sample_organis… Saccharom… Saccharom… Saccharom… Saccharom… Saccharom…
##  8 !Sample_charact… pho80 loc… pho80 loc… pho80 loc… pho80 loc… pho80 loc…
##  9 !Sample_charact… pho2 locu… pho2 locu… pho2 locu… pho2 locu… pho2 locu…
## 10 !Sample_charact… pho4 locu… pho4 locu… pho4 locu… pho4 locu… pho4 locu…
## # … with 35 more rows, and 31 more variables: GSM2577562 <chr>,
## #   GSM2577563 <chr>, GSM2577564 <chr>, GSM2577565 <chr>, GSM2577566 <chr>
,
## #   GSM2577567 <chr>, GSM2577568 <chr>, GSM2577569 <chr>, GSM2577570 <chr>
,
## #   GSM2577571 <chr>, GSM2577572 <chr>, GSM2577573 <chr>, GSM2577574 <chr>
,
## #   GSM2577575 <chr>, GSM2577576 <chr>, GSM2577577 <chr>, GSM2577578 <chr>
,
## #   GSM2577579 <chr>, GSM2577580 <chr>, GSM2577581 <chr>, GSM2577582 <chr>
,
## #   GSM2577583 <chr>, GSM2577584 <chr>, GSM2577585 <chr>, GSM2577586 <chr>
,
## #   GSM2577587 <chr>, GSM2577588 <chr>, GSM2577589 <chr>, GSM2577590 <chr>
,
## #   GSM2577591 <chr>, GSM2577592 <chr>
```

We will analyze this data set to see which transcriptome profiles exhibit a similar repression of the PsAvh110 most-repressed genes. This data set contains count data, which must be analyzed to generate log ratios and adj. p-values.

```
# Download sequence count data matrix
countData <- read_csv("https://www.ncbi.nlm.nih.gov/geo/download/?acc=GSE9779
9&format=file&file=GSE97799_ScerRNAseq_count.csv.gz")
```

Effect of pho4 deletion in pho80 deletion background

To identify genes differentially expressed in the absence of pho4, we first select the desired samples from the count data file: ScPHO4 PHO2 (A1a, A2a) vs. pho4_KO PHO2 (C1a, C2a). After setting the factors for each sample, we then construct an appropriate data set and perform differential gene expression analysis using DESeq. After resetting the basal condition for comparisons, we extract the results table with log2 fold changes and p-values. Finally, a summary of the results is presented.

```
countData_pho4 <- countData %>%
  dplyr::select("A1a", "A2a", "C1a", "C2a")

condition <- factor(c("PHO4", "PHO4", "pho4delta", "pho4delta"))
dds <- DESeqDataSetFromMatrix(countData = countData_pho4,
                              colData = DataFrame(condition),
                              design = ~ condition)

dds <- DESeq(dds) #create large DESeqDataSet
dds$condition <- relevel(dds$condition, "PHO4") #set basal condition for comp
arisons
```

```
res <- results(dds) #extract results from large dataset
summary(res) #show summary of results
```

```
##
## out of 5954 with nonzero total read count
## adjusted p-value < 0.1
## LFC > 0 (up)       : 376, 6.3%
## LFC < 0 (down)     : 331, 5.6%
## outliers [1]       : 0, 0%
## low counts [2]     : 230, 3.9%
## (mean count < 1)
## [1] see 'cooksCutoff' argument of ?results
## [2] see 'independentFiltering' argument of ?results
```

Of the nearly 6000 yeast genes with detectable expression, over 700 are differentially expressed (approximately equally up- and down-regulated) in the pho4 mutant as compared to the PHO4 strain in the pho80 deletion background.

We next construct a simple matrix with lfc data for further analysis.

```
GSE97799_pho4 <- countData %>%
  dplyr::select("ORF") %>%
  bind_cols(as_tibble(res)) %>%
  dplyr::select(ORF, pho4 = "log2FoldChange")

GSE97799_longer <- GSE97799_pho4 %>%
  pivot_longer(-ORF, names_to = "Sample_geo_accession", values_to = "log_rati
o")
```

To compare the pho4 transcriptome profile to the PsAvh110-repressed gene set, we rank each gene's expression level, extract the rankings of the PsAvh110-repressed genes, and compute the mean of log-transformed rankings for these genes.

```
GSE97799_rank <- GSE97799_pho4  %>%
  mutate_if(is.numeric, funs(min_rank))
```

```
## Warning: `funs()` is deprecated as of dplyr 0.8.0.
## Please use a list of either functions or lambdas:
##
##   # Simple named list:
##   list(mean = mean, median = median)
##
##   # Auto named with `tibble::lst()`:
##   tibble::lst(mean, median)
##
##   # Using lambdas
##   list(~ mean(., trim = .2), ~ median(., na.rm = TRUE))
## This warning is displayed once every 8 hours.
## Call `lifecycle::last_warnings()` to see where this warning was generated.
```

```
GSE97799_rank_BottomGenes <- GSE97799_rank %>% filter(ORF %in% BottomGenes)

GSE97799_mean_logrank_BottomGenes <- GSE97799_rank_BottomGenes %>%
  pivot_longer(cols = -1, names_to = "Sample_geo_accession", values_to = "Sam
ple_rank") %>%
    filter(!is.na(Sample_rank)) %>%
  group_by(Sample_geo_accession) %>%
  summarise(mean_logrank = 10^mean(log10(Sample_rank)), n = n()) %>%
  filter(n > 2) %>%
  arrange(mean_logrank)

## `summarise()` ungrouping output (override with `.groups` argument)

GSE97799_mean_logrank_BottomGenes

## # A tibble: 1 x 3
##   Sample_geo_accession mean_logrank     n
##   <chr>                       <dbl> <int>
## 1 pho4                         3.63     5
```

To examine the pho4 data more closely, we display the rankings of the PsAvh110-repressed genes.

```
GSE97799_pho4_BG <- GSE97799_longer %>%
  filter(ORF %in% BottomGenes)
GSE97799_pho4_NBG <- GSE97799_longer %>%
  filter(!ORF %in% BottomGenes)

Plot_data <- GSE97799_rank_BottomGenes %>%
  pivot_longer(-ORF, names_to = "Sample_geo_accession", values_to = "Rank_in_
sample") %>%
  left_join(GSE97799_longer, by = c("ORF", "Sample_geo_accession"))

Plot_data %>%
  ggplot(aes(x=Sample_geo_accession, y=log_ratio)) +
  geom_point(data=filter(GSE97799_longer, !ORF %in% BottomGenes,
                         log_ratio < 0),
             na.rm = TRUE,
             position=position_jitter(0.1,0), color="black", alpha = 0.2) +
  geom_label(aes(label=Rank_in_sample, color = ORF),
             position = position_dodge2(width = 0.3),
            label.padding = unit(0.05, "lines"),
            na.rm = TRUE,
            show.legend = FALSE) +
  geom_label(data = GSE97799_mean_logrank_BottomGenes,
            aes(label=signif(mean_logrank,3), y=min(Plot_data$log_ratio, na.r
m = TRUE)),
            nudge_y = -0.2,
            label.padding = unit(0.10, "lines"),
            show.legend = FALSE) +
```

```
  geom_point(aes(color = ORF), size=NA, na.rm = TRUE) + # to replace geom_lab
el legend symbols
  labs(x = NULL,
       y = "log2 fold change (mutant/control)") +
  theme_classic(base_size = 13) +
  theme(axis.text.x=element_text(angle=30,hjust=1)) +
  scale_y_continuous(limits = c(min(Plot_data$log_ratio)-1, 0)) +
  scale_color_discrete(name = "Gene name", labels = BottomGeneTable$Gene_name
) +
  guides(colour=guide_legend(override.aes=list(size=3)))
```

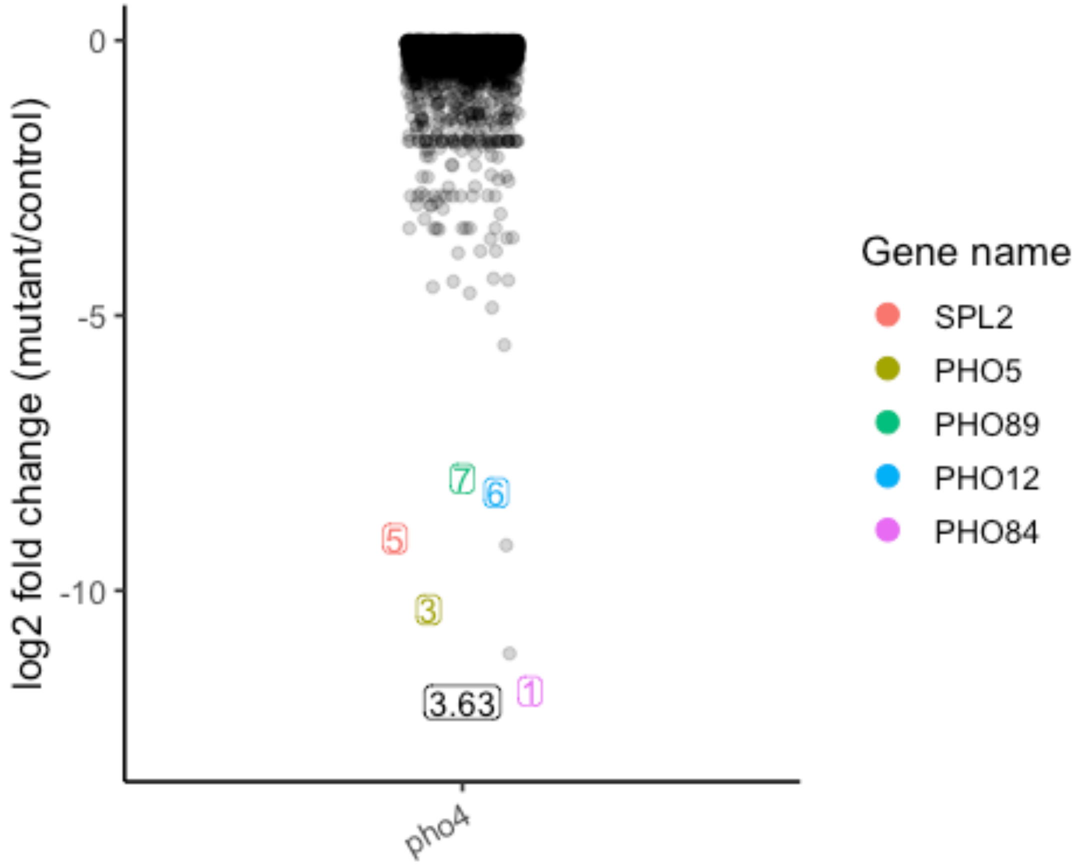

Figure S2-3. Expression levels and sample ranks of the PsAvh110-repressed genes in the pho4 mutant. The relative expression levels of the five PsAvh110-repressed genes are indicated by a colored box labeled with their rank position. The relative expression levels of all other yeast genes are indicated by gray dots. The mean of the gene ranks (after removing missing data) and the mutant genotype of each sample is indicated below.

Strikingly, the five PsAvh110 genes are among the seven most down-regulated genes in the pho4 vs. PHO4 comparison. What are the other genes in the bottom 10?

```
GSE97799_rank %>%
  arrange(pho4) %>%
```

```r
  left_join(GeneTable, by="ORF") %>%
  print()
```

```
## # A tibble: 6,383 x 3
##    ORF      pho4 Gene_name
##    <chr>   <int> <chr>
##  1 YML123C     1 PHO84
##  2 YFR034C     2 PHO4
##  3 YBR296C     3 PHO89
##  4 YDR281C     4 PHM6
##  5 YBR093C     5 PHO5
##  6 YHR215W     6 PHO12
##  7 YHR136C     7 SPL2
##  8 YAR071W     8 PHO11
##  9 YCR098C     9 GIT1
## 10 YPL165C    10 SET6
## # … with 6,373 more rows
```

The genes most down-regulated in the pho4 deletion strain includes the 5 PsAvh110 most-repressed genes, as well as PHO4 (obviously) and PHM6.

What is the overlap between the bottom pho4 genes and the bottom PsAvh110 genes? We compare the ranks of the bottom 20 pho4 genes in the PsAvh110 sample and vice-versa.

```r
GSE97799_rank %>%
  filter(pho4 < 21) %>%
  arrange(pho4) %>%
  left_join(GeneTable, by="ORF") %>%
  left_join(dplyr::select(EMTAB9566_rank, ORF = "GeneID", EMTAB9566_rank = "S
ample_rank"), by="ORF")
```

```
## # A tibble: 20 x 4
##    ORF      pho4 Gene_name EMTAB9566_rank
##    <chr>   <int> <chr>              <int>
##  1 YML123C     1 "PHO84"                3
##  2 YFR034C     2 "PHO4"              1202
##  3 YBR296C     3 "PHO89"                1
##  4 YDR281C     4 "PHM6"                68
##  5 YBR093C     5 "PHO5"                 4
##  6 YHR215W     6 "PHO12"                5
##  7 YHR136C     7 "SPL2"                 2
##  8 YAR071W     8 "PHO11"              361
##  9 YCR098C     9 "GIT1"                70
## 10 YPL165C    10 "SET6"              4546
## 11 snR17a     11 ""                    NA
## 12 YIL169C    12 ""                   325
## 13 SCR1       13 ""                    NA
## 14 snR190     14 ""                    NA
## 15 YPL019C    15 "VTC3"                22
## 16 snR35      16 ""                    NA
## 17 YJL028W    17 ""                  3141
```

```
## 18 snR7-S     17 ""                    NA
## 19 YBR050C    19 "REG2"                73
## 20 YPL018W    20 "CTF19"               45
```

```
EMTAB9566_rank %>%
  filter(Sample_rank < 21) %>%
  arrange(Sample_rank) %>%
  dplyr::rename(ORF = "GeneID", EMTAB9566_rank = "Sample_rank") %>%
  left_join(GeneTable, by="ORF") %>%
  left_join(GSE97799_rank, by="ORF")
```

```
## # A tibble: 20 x 4
##    ORF     EMTAB9566_rank Gene_name  pho4
##    <chr>            <int> <chr>     <int>
##  1 YBR296C              1 "PHO89"       3
##  2 YHR136C              2 "SPL2"        7
##  3 YML123C              3 "PHO84"       1
##  4 YBR093C              4 "PHO5"        5
##  5 YHR215W              5 "PHO12"       6
##  6 YOR385W              6 ""          876
##  7 YCR106W              7 "RDS1"      382
##  8 YLR136C              8 "TIS11"     160
##  9 YHL028W              9 "WSC4"     2488
## 10 YCR107W             10 "AAD3"      437
## 11 YJL102W             11 "MEF2"     4424
## 12 YDR123C             12 "INO2"     3895
## 13 YGL255W             13 "ZRT1"     5734
## 14 YMR251W             14 "GTO3"      162
## 15 YOL016C             15 "CMK2"      556
## 16 YER020W             16 "GPA2"      412
## 17 YOR382W             17 "FIT2"     4422
## 18 YBR285W             18 ""         5332
## 19 YMR011W             19 "HXT2"      337
## 20 YNL037C             20 "IDH1"     3639
```

Of the 20 genes most down-regulated in the pho4 deletion mutant, many are significantly down-regulated in the PsAvh110 sample. These include the 5 most down-regulated genes, as well as PHM6, GIT1, VTC3, REG2, and CTF19.

However, by examining both tables, we see that the overlap in down-regulated genes, while extensive, is not complete.

APPENDIX: Estimate p-value of mean log rank using simulation

To estimate the p-values of a mean log rank, we perform 1,000,000 simulations to randomly assign 5 rank values (from 1 to the total number of yeast genes, 6307) and then compute the mean log rank. The Bonferroni-adjusted p-value of 0.001 is calculated by the number of samples (264 in the GSE5499 data set).

```
rmean_logrank <- vector(mode = "numeric")

antilog<-function(lx,base) {
  lbx<-lx/log(exp(1),base=base)
  result<-exp(lbx)
  result
}

ngenes <- 6307

for(i in 1:1000000) {
  rmean_logrank[i] <- runif(5, min = 0, max = ngenes) %>%
    ceiling %>% log10() %>% mean() %>% antilog(10)
}

quantile(rmean_logrank, probs = c(0, 0.001, 0.01, 0.1, 1, 5, 50, 100)/100)

##         0%     0.001%      0.01%       0.1%         1%          5%         5
0%
##   74.79465  109.29228  199.85811  343.45149  626.75838 1013.27077 2478.103
66
##       100%
## 6109.15743

nsamples = 264

# mean rank cut-off for Bonferroni adj. p-value < 0.001
quantile(rmean_logrank, probs = 0.001/nsamples)

## 0.0003787879%
##      92.15817
```
